# Mol­ecular structure of *fac*-[Mo(CO)_3_(DMSO)_3_]

**DOI:** 10.1107/S2056989021004448

**Published:** 2021-04-30

**Authors:** Benedict J. Elvers, Christian Fischer, Carola Schulzke

**Affiliations:** a Institut für Biochemie, Universität Greifswald, 4 Felix-Hausdorff-Straβe, 17489 Greifswald, Germany

**Keywords:** crystal structure, molybdenum, carbonyl complexes, solvent complexes, *fac*-coordination

## Abstract

The title compound, tri­carbonyl­tris­(dimethyl sulfoxide)­molybdenum, [Mo(C_2_H_6_OS)_3_(CO)_3_] or *fac*-[Mo(CO)_3_(DMSO)_3_], crystallizes in the triclinic space group *P*  with two mol­ecules in the unit cell. The geometry around the central molybdenum is slightly distorted octa­hedral and the *facial* isomer is found exclusively. The packing within the crystal is stabilized by three-dimensional non-classical inter­molecular hydrogen-bonding contacts between individual methyl substituents of di­methyl sulfoxide and the oxygen atoms of either another di­methyl sulfoxide or a carbonyl ligand on adjacent complex mol­ecules. The observed bond lengths in the carbonyl ligands and between carbonyl carbon atoms and molybdenum are correlated to the observed FT–IR bands for the carbonyl stretches and compared to respective metrical parameters of related complexes.

## Abstract 2

The title compound *fac*-[Mo(CO)_3_(DMSO)_3_] was synthesized and crystallized as an alternative starting material for the more frequently used *fac*-[Mo(CH_3_CN)_3_(CO)_3_]. The DMSO complex was first reported in 1959 and apparently never mentioned again afterwards. Its crystal structure remained unknown to date. The observed metrical parameters of the now presented structural X-ray diffraction analysis are correlated to FT–IR data and compared to related complexes. Packing patterns and inter­molecular non-classical hydrogen-bonding inter­actions are discussed.

## Chemical context   

[Mo(CO)_6_] is a commercially available starting material that is easy to handle. It is, however, not particularly reactive. In order to facilitate quicker and/or more complete reactions, it can be activated by replacing some of the CO ligands by solvent ligands. This is often done with aceto­nitrile, which results in *fac*-[Mo(CH_3_CN)_3_(CO)_3_] complexes. Other examples comprise, for instance, [Mo(CO)_5_(THF)] (THF = tetra­hydro­furane), [Mo(CO)_4_(nbd)] (nbd = norbornadiene) and *fac*-[Mo(CO)_3_(DMF)_3_] (DMF = di­methyl formamide) (Wieland & van Eldik, 1991 ▸; Mukerjee *et al.* 1988 ▸; Villanueva *et al.*, 1996 ▸). Depending on the co-ligand, the stability and reactivity of the resultant complex can be fine-tuned. It was, for example, previously emphasized that the pyridine complexes surpass aceto­nitrile complexes in reactivity (Kuhl *et al.*, 2000 ▸). In cases where the carbonyl ligands are supposed to be retained, stronger carbon­yl–metal inter­actions and very weak metal–co-ligand inter­actions are preferred. In cases where the carbonyl ligands shall also be replaced, the opposite is true. The grade of activation is reflected in the C≡O bond lengths and the Mo—C_carbon­yl_ bond lengths. For the former, infrared spectroscopy provides an easy way to probe the strength of the bond between carbon and oxygen with stretching vibration bands in a normally not populated region of the infrared wavenumber range (around 2000 cm^−1^). This bond strength depends directly on the metal–carbon inter­action as the stronger the metal carbon bond, the weaker the carbon–oxygen bond becomes (Elschenbroich, 2003 ▸) and these again depend on the strengths of the *trans*-located co-ligand-to-metal inter­actions. A short and strong C≡O bond is, hence, indicative of only weak carbonyl metal–ligand inter­actions and concomitantly impaired complex stability. FT–IR therefore constitutes a particularly helpful assessment tool, in particular in cases where no crystal structure is available. On the other hand, it is also quite useful to combine both methods, if possible, for validation purposes and adding reliability to future spectroscopic evaluation of related species. In the course of synthesizing molybdenum–carbonyl complexes as starting materials and in a search for the optimum balance between reactivity and stability, various solvent complexes were tested in our group. During these experiments, DMSO was considered beneficial and the title complex *fac*-[Mo(CO)_3_(DMSO)_3_] was prepared and crystallized. This complex was first reported in the literature in 1959 (Hieber *et al.*, 1959 ▸), but its crystal structure remained, apparently, elusive to date. Notably, it also appears that since then the complex has never been mentioned again. As very nice and suitable crystals of the title compound were obtained, an X-ray diffraction structural analysis was carried out. The respective high-quality results, along with the signatory carbonyl FT–IR stretch bands are presented here.
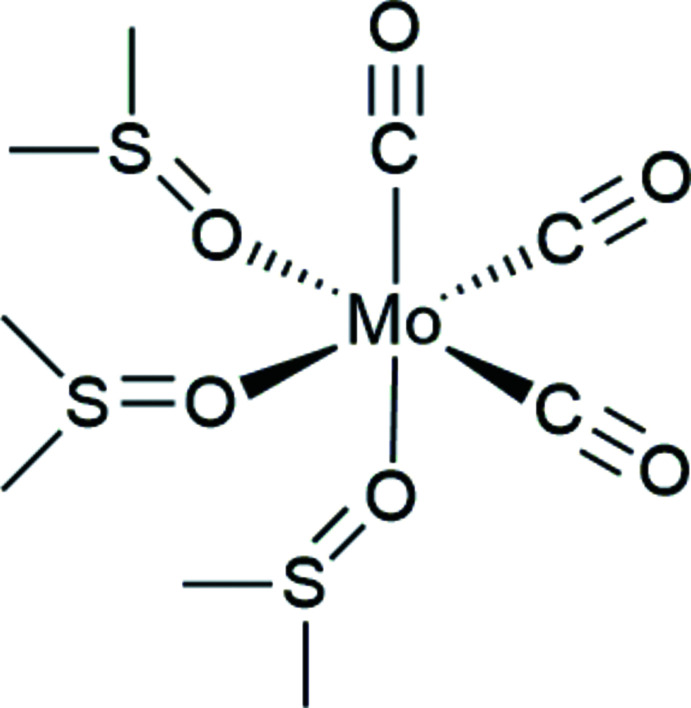


## Structural commentary   

**Figure 1:**
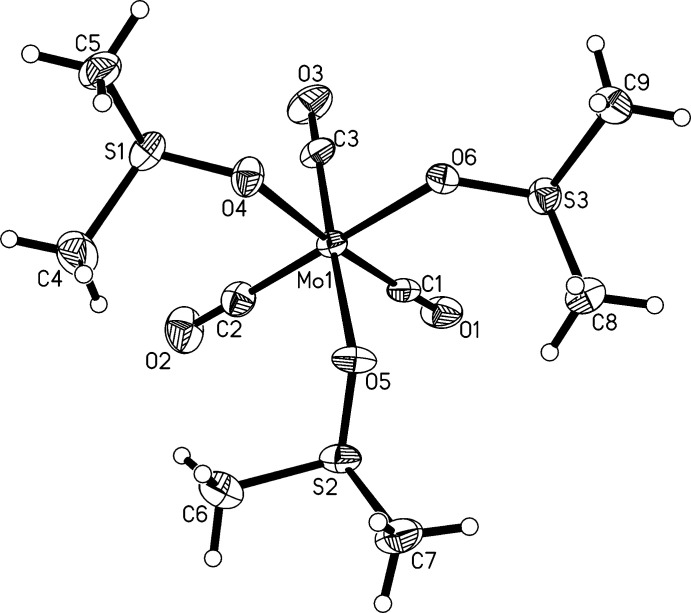
Mol­ecular structure of *fac*-[Mo(CO)_3_(DMSO)_3_] with ellipsoids at the 50% level.

*fac*-[Mo(CO)_3_(DMSO)_3_] crystallizes in the triclinic space group *P* 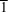. The asymmetric unit represents the entire mol­ecule (Fig. 1 ▸) while *Z* = 2. The central zero-valent molybdenum is coordinated in a *facial* fashion by three neutral di­methyl sulfoxide and three neutral carbonyl ligands, *i.e.* it is embraced by a C_3_O_3_ donor set. The coordination geometry of the complex is essentially octa­hedral, showing an almost perfect Bailar twist angle (Wentworth, 1972 ▸) of 59.08°. The average *cis*-donor—Mo—donor angle between the three coordinated DMSO mol­ecules is, at approximately 79°, slightly more acute compared to that of the carbonyl ligands, at approximately 84° despite di­methyl sulfoxide being considerably more bulky. The three *trans* angles across molybdenum range from 173.76 (16) to 178.08 (18)°, indicating a slight distortion from ideal octa­hedral geometry.

**Figure 2:**
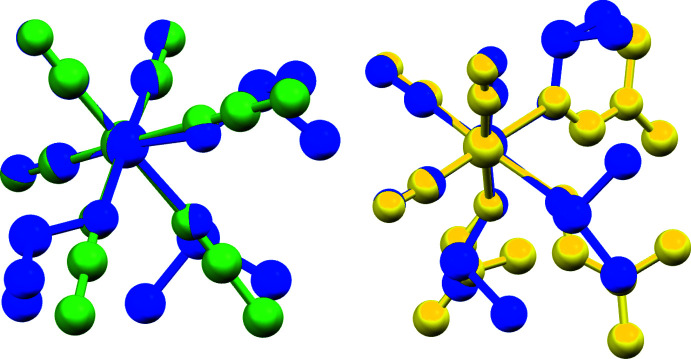
Structural overlay between *fac*-[Mo(CO)_3_(DMSO)_3_] in blue with *fac*-[Mo(CH_3_CN)_3_(CO)_3_] (refcode: IZUQAV; Antonini *et al.*, 2004 ▸) in green (left) and with *fac*-[Mo(CO)_3_(DMF)_3_] (refcode: WAJWIN; Pasquali *et al.*, 1992 ▸) in yellow (right) generated with *Mercury* 2020.3.0 (Macrae *et al.*, 2020 ▸).

The structures of the title compound and those of chemic­ally very closely related *fac*-[Mo(CH_3_CN)_3_(CO)_3_] (refcode: IZUQAV; Antonini *et al.*, 2004 ▸) and *fac*-[Mo(CO)_3_(DMF)_3_] (refcode: WAJWIN; Pasquali *et al.*, 1992 ▸) are, as expected, quite similar in the immediate coordination sphere surrounding molybdenum, which is also evident from the overlaid mol­ecular structures (Fig. 2 ▸). Still, some specifics in the metrical parameter details in the individual species are quite notable.

**Figure 3:**
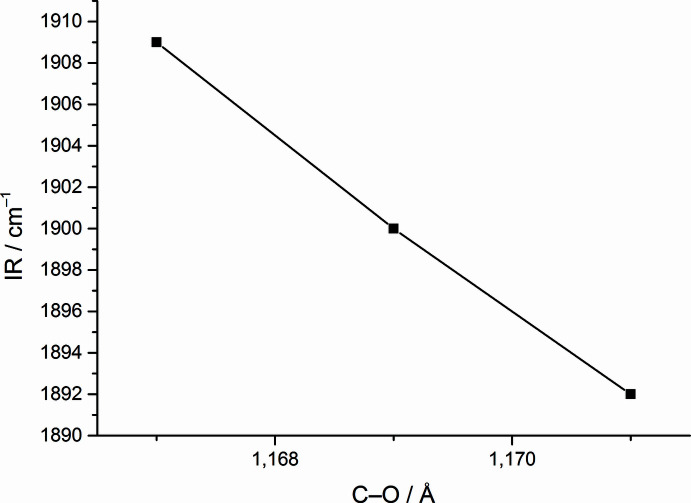
Correlation between the highest energy infrared band for the C—O stretching vibration and the shortest of the observed C—O bonds in the mol­ecular structures of *fac*-[Mo(CO)_3_(DMSO)_3_], *fac*-[Mo(CH_3_CN)_3_(CO)_3_] (refcode: IZUQAV; Antonini *et al.*, 2004 ▸) and *fac*-[Mo(CO)_3_(DMF)_3_] (refcode: WAJWIN; Pasquali *et al.*, 1992 ▸).

In particular the C—O and Mo—C distances are inter­esting when compared to those of *fac*-[Mo(CH_3_CN)_3_(CO)_3_] and *fac*-[Mo(CO)_3_(DMF)_3_]. Whereas the average C—O bond length in *fac*-[Mo(CO)_3_(DMSO)_3_] is 1.170 (6) Å and the average Mo—C distance is 1.911 (5) Å, in *fac*-[Mo(CH_3_CN)_3_(CO)_3_] the average C—O and Mo—C distances are 1.167 and 1.923 Å, respectively. In *fac*-[Mo(CO)_3_(DMF)_3_], these values are 1.172 Å (C—O) and 1.909 Å (Mo—C). The complexes with the O-donor solvent coordination exhibit longer C—O and shorter Mo—C distances, which is indicative of stronger bonds between carbonyl and molybdenum than in the case of the N-donor solvent. At the same time, this suggests that the share of electron density between molybdenum and coordinated solvent is decreased in the case of O-donor solvents and increased in the case of the N-donor solvent. This is also reflected in the reported IR data. In the case of aceto­nitrile, two C—O bands are reported, and for the other two complexes, three. In perfectly octa­hedral symmetry, only two bands would be expected (Elschenbroich, 2003 ▸). The presence of three bands therefore indicates a distortion of the complex from perfect symmetry. The comparison of the highest energy infrared bands with the shortest observed C—O bond lengths in these three species reveals a perfect correlation (Fig. 3 ▸).

The O-donor solvents, therefore, appear to be slightly better suited for those reactions in which the carbonyl ligands are supposed to be retained on the metal, while the co-ligands are more labile. In the case of *fac*-[Mo(CO)_3_(DMSO)_3_], it was observed that the complex is very sensitive to moisture, for instance, which supports the anti­cipated facile exchange of the coordinated solvents.

**Figure 4:**
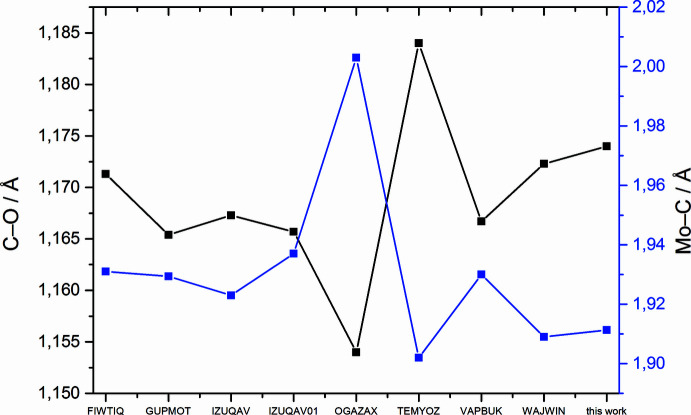
Average C—O and Mo—C distances in the mol­ecular structures of neutral bis­leptic tris­carbonyl molybdenum(0) complexes with oxygen or nitro­gen donor co-ligands.

When larger co-ligands are also included in the C—O and Mo—C bond-length analysis, these observations are generally confirmed (Fig. 4 ▸). Only the structures with methyl-pyridine (refcode: TEMYOZ; Schut *et al.*, 1996 ▸) and pyrazole (refcode: OGAZAX; Ardizzoia *et al.*, 2002 ▸) exhibit somewhat extreme values with a particularly short and strong C—O bond in the latter and an exceptionally long and weak C—O bond in the former, which even surpasses the effect of the O-donor co-ligands. The other considered structures comprise a second one with aceto­nitrile (refcode: IZUQAVO1; Sala *et al.*, 2018 ▸), one with propio­nitrile (refcode: FIWTIQ; Hering *et al.*, 2014 ▸), one with thio­phene-aceto­nitrile (refcode: VAPBUK; Baker *et al.*, 2003 ▸), and one with pyridine (refcode: GUPMOT, Kuhl *et al.*, 2000 ▸).

## Supra­molecular features   

**Figure 5:**
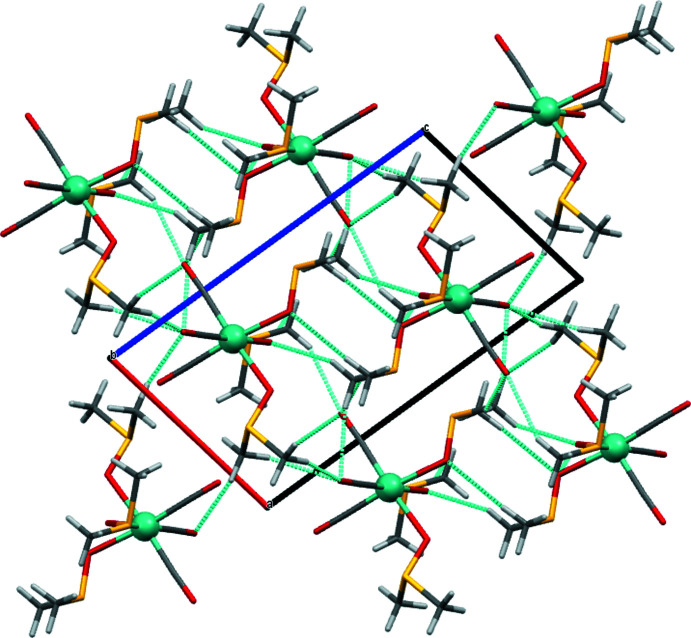
Crystal packing viewed along the *b* axis with hydrogen-bonding contacts shown as light blue dashed lines, generated with *Mercury* 2020.3.0 (Macrae *et al.*, 2020 ▸).

**Figure 6:**
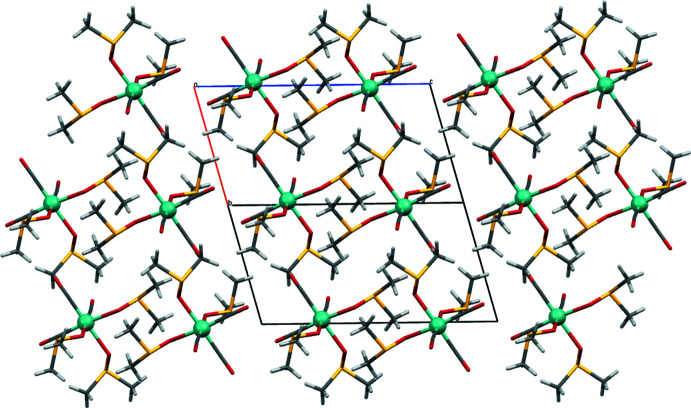
Crystal packing viewed along the *ab* diagonal with the *c* axis in horizontal alignment along which the orientations of the complex mol­ecules alternate, generated with *Mercury* 2020.3.0 (Macrae *et al.*, 2020 ▸).

The unit cell is relatively small and contains only two mol­ecules. Non-classical hydrogen-bonding contacts stabilize the crystal packing (Table 1 ▸). All hydrogen atoms are part of methyl groups and these are thereby the only available donors. Oxygen atoms of DMSO (O5, O6) and of carbonyl ligands (O1, O3) serve as hydrogen-bonding acceptors. Hydrogen bonding within the unit cell involves exclusively DMSO. Hydrogen bonding between unit cells is exclusively between DMSO and carbonyl oxygen atoms (Fig. 5 ▸). The orientations of the mol­ecules strictly alternate in the *c*-axis direction, as is evident when viewed along the *ab* diagonal (Fig. 6 ▸) while they are identical to those of their neighbours in the *a*- and *b*-axis directions.

## Database survey   

A search of the CSD database with *ConQuest* (Bruno *et al.*, 2002 ▸) for bis­leptic tris­carbonyl molybdenum(0) complexes and three neutral co-ligands with N, O, S or P donor atoms results, in addition to the eight known mol­ecular structures with oxygen or nitro­gen donors, which are already discussed in the structural commentary, only in species with phospho­rous donor atoms. These are structures with the refcodes DUSHAA (Tarassoli *et al.*, 1986 ▸), DUSHAA10 (Chen *et al.*, 1986 ▸), JEWPIL (Nakazawa *et al.*, 2006 ▸), KETQIJ (Campbell *et al.*, 1999 ▸), KOBSIE (Fukumoto & Nakazawa, 2008 ▸), LALSEW (Willey *et al.*, 1993 ▸), NIPTAH and NIPTEL (Alyea *et al.*, 1997 ▸), NITFOM (Tallis *et al.*, 2008 ▸), SANMOJ (Bent *et al.*, 1989 ▸), SANMOJ10 (Bent *et al.*, 1990 ▸), TAWNIO (Edwards *et al.*, 1996 ▸), TIRYUP (Thirupathi *et al.*, 2007 ▸), YAZSAT and YAZSIB (Kang *et al.*, 1994 ▸), YAZSAT10 and YAZSIB10 (Hockless *et al.*, 1996 ▸), YEPWAR (Fischer *et al.*, 1994 ▸), and ZEXCIO (Alyea *et al.*, 1995 ▸). The average C—O bond lengths in the mol­ecular structures with phospho­rous donor atoms range from 1.141 Å (KOBSIE) to 1.228 Å (DUSHAA). This means that both shorter as well as longer bonds are observed in the P-donor species than in the O- and N-donor complexes. In total only 27 examples of complexes are found in the database that meet the search criteria. Considering the simplicity of the complexes this is a surprisingly small number.

## Synthesis and crystallization   

A tempered reaction vessel (293 K) was charged with [Mo(CO)_6_] (165 mg, 0.625 mmol, 1 eq.) and the atmosphere was replaced by argon. 10 ml of absolute tetra­hydro­furane (THF) and 0.2 ml of absolute di­methyl sulfoxide (DMSO, 2.82 mmol, 4.5 eq.) were added and the reaction vessel was irradiated for 2 h with HPM13 and HPA1200 halogen lamps as in previously described related activation procedures (Elvers *et al.*, 2019 ▸). The resulting yellow solution was transferred anaerobically into a Schlenk flask and dried *in vacuo*. The golden-yellow solid precipitate was re-dissolved in THF and layered with *n*-hexane. Light-yellow, prismatic crystals of the title compound formed after three days of slow diffusion. Yield: 66.9% (160.7 mg, 0.418 mmol). IR (as KBr pellet given in cm^−1^): 2260 (*w*, *br*); 1890 (*s*); 1750 (*s*); 1724 (*s*); 1308 (*sh*); 1246 (*s*); 1153 (*s*); 1020 (*sh*); 978 (*s*); 824 (*s*); 760 (*s*) (*w* = weak/*s* = strong/*sh* = shoulder/*br* = broad) .

## Refinement   

Crystal data, data collection and structure refinement details are summarized in Table 2 ▸. All hydrogen atoms belong to methyl substituents. They were attached to their parent atom in calculated positions based on the presence of electron density (HFIX 137) and treated as riding with *U*_iso_(H) = 1.5 *U*_eq_(C). One reflection was omitted from the refinement as a clear outlier. *WinGX* was used as GUI for solving and refining the structure (Farrugia, 2012 ▸).

## Supplementary Material

Crystal structure: contains datablock(s) I. DOI: 10.1107/S2056989021004448/zq2262sup1.cif

Structure factors: contains datablock(s) I. DOI: 10.1107/S2056989021004448/zq2262Isup2.hkl

CCDC reference: 2080083

Additional supporting information:  crystallographic information; 3D view; checkCIF report

## Figures and Tables

**Table 1:** Hydrogen-bond geometry (Å, °)

*D*—H⋯*A*	*D*—H	H⋯*A*	*D*⋯*A*	*D*—H⋯*A*
C5—H5*C*⋯O1^i^	0.98	2.52	3.461 (7)	161
C4—H4*C*⋯O3^ii^	0.98	2.60	3.503 (8)	154
C7—H7*C*⋯O1^ii^	0.98	2.51	3.419 (7)	154
C9—H9*C*⋯O5^iii^	0.98	2.45	3.239 (7)	138
C4—H4*B*⋯O3^iv^	0.98	2.38	3.334 (8)	165
C9—H9*A*⋯O1^v^	0.98	2.59	3.328 (7)	132
C9—H9*B*⋯O3^v^	0.98	2.52	3.469 (7)	163
C7—H7*B*⋯O6^vi^	0.98	2.55	3.398 (6)	144

Symmetry codes: (i) x+1, y-1, z; (ii) x+1, y, z; (iii) -x+1, -y+1, -z+1; (iv) -x+1, -y+1, -z; (v) -x, -y+1, -z+1; (vi) x, y+1, z.

**Table 2:** Experimental details

Crystal data	
Chemical formula	[Mo(C_2_H_6_OS)_3_(CO)_3_]
*M*_r_	414.35
Crystal system, space group	Triclinic, *P*\overline{1}
Temperature (K)	170
*a*, *b*, *c* (Å)	8.2027 (16), 8.4059 (17), 13.465 (3)
α, β, γ (°)	78.58 (3), 75.69 (3), 63.94 (3)
*V* (Å^3^)	803.8 (4)
*Z*	2
Radiation type	Mo *K*α
μ (mm^−1^)	1.22
Crystal size (mm)	0.27 × 0.09 × 0.04

Data collection	
Diffractometer	Stoe IPDS2T
Absorption correction	Numerical face indexed (*X-RED32* and *X-SHAPE*; Stoe & Cie, 2010 ▸)
*T*_min_, *T*_max_	0.909, 0.989
No. of measured, independent and observed [*I* > 2σ(*I*)] reflections	8903, 4417, 3408
*R*_int_	0.054
(sin θ/λ)_max_ (Å^−1^)	0.693

Refinement	
*R*[*F* ^2^ > 2σ(*F* ^2^)], *wR*(*F* ^2^), *S*	0.053, 0.135, 1.07
No. of reflections	4417
No. of parameters	178
H-atom treatment	H-atom parameters constrained
Δρ_max_, Δρ_min_ (e Å^−3^)	1.10, −2.32

Computer programs: *X-AREA* (Stoe & Cie, 2016 ▸), *SHELXT2018* (Sheldrick, 2015*a* ▸), *SHELXL2018/3* (Sheldrick, 2015*b* ▸) and *XP* (Sheldrick, 2008 ▸), *CIFTAB* (Sheldrick, 2008 ▸).

## supplementary crystallographic information

### Crystal data

**Table:**

[Mo(C_2_H_6_OS)_3_(CO)_3_]	*Z* = 2
*M**_r_* = 414.35	*F*(000) = 420
Triclinic, *P*1	*D*_x_ = 1.712 Mg m^−^^3^
*a* = 8.2027 (16) Å	Mo *K*α radiation, λ = 0.71073 Å
*b* = 8.4059 (17) Å	Cell parameters from 8904 reflections
*c* = 13.465 (3) Å	θ = 6.5–59.0°
α = 78.58 (3)°	µ = 1.22 mm^−^^1^
β = 75.69 (3)°	*T* = 170 K
γ = 63.94 (3)°	Needle, yellow
*V* = 803.8 (4) Å^3^	0.27 × 0.09 × 0.04 mm

### Data collection

**Table:**

Stoe IPDS2T diffractometer	4417 independent reflections
Radiation source: fine-focus sealed tube	3408 reflections with *I* > 2σ(*I*)
Detector resolution: 6.67 pixels mm^-1^	*R*_int_ = 0.054
ω scans	θ_max_ = 29.5°, θ_min_ = 3.3°
Absorption correction: numerical face indexed (X-Red32 and X-Shape; Stoe & Cie, 2010)	*h* = −11→11
*T*_min_ = 0.909, *T*_max_ = 0.989	*k* = −11→11
8903 measured reflections	*l* = −16→18

### Refinement

**Table:**

Refinement on *F*^2^	Primary atom site location: dual
Least-squares matrix: full	Secondary atom site location: difference Fourier map
*R*[*F*^2^ > 2σ(*F*^2^)] = 0.053	Hydrogen site location: inferred from neighbouring sites
*wR*(*F*^2^) = 0.135	H-atom parameters constrained
*S* = 1.07	*w* = 1/[σ^2^(*F*_o_^2^) + (0.0653*P*)^2^ + 1.7985*P*] where *P* = (*F*_o_^2^ + 2*F*_c_^2^)/3
4417 reflections	(Δ/σ)_max_ < 0.001
178 parameters	Δρ_max_ = 1.10 e Å^−^^3^
0 restraints	Δρ_min_ = −2.32 e Å^−^^3^

### Special details

**Table:**

Geometry. All esds (except the esd in the dihedral angle between two l.s. planes) are estimated using the full covariance matrix. The cell esds are taken into account individually in the estimation of esds in distances, angles and torsion angles; correlations between esds in cell parameters are only used when they are defined by crystal symmetry. An approximate (isotropic) treatment of cell esds is used for estimating esds involving l.s. planes.

### Fractional atomic coordinates and isotropic or equivalent isotropic displacement parameters (Å^2^)

**Table:**

	*x*	*y*	*z*	*U*_iso_*/*U*_eq_	
Mo1	0.26439 (5)	0.70392 (5)	0.25843 (3)	0.01937 (12)	
S1	0.64463 (15)	0.46716 (16)	0.11748 (9)	0.0276 (3)	
S2	0.31315 (15)	1.05860 (15)	0.28585 (10)	0.0267 (2)	
S3	0.16962 (15)	0.60278 (16)	0.50846 (9)	0.0259 (2)	
O1	−0.1521 (5)	0.9399 (5)	0.3125 (3)	0.0366 (9)	
O2	0.2294 (6)	0.9145 (6)	0.0427 (3)	0.0437 (10)	
O3	0.1020 (5)	0.4935 (6)	0.1823 (3)	0.0425 (10)	
O4	0.5662 (4)	0.5143 (5)	0.2275 (3)	0.0261 (7)	
O5	0.3793 (5)	0.8603 (4)	0.3182 (3)	0.0276 (7)	
O6	0.3177 (4)	0.5503 (4)	0.4125 (2)	0.0241 (7)	
C1	0.0078 (6)	0.8542 (6)	0.2948 (4)	0.0244 (9)	
C2	0.2447 (6)	0.8356 (6)	0.1249 (4)	0.0277 (10)	
C3	0.1697 (6)	0.5673 (6)	0.2123 (4)	0.0269 (9)	
C4	0.7296 (8)	0.6302 (8)	0.0556 (5)	0.0441 (14)	
H4A	0.625940	0.747086	0.050873	0.066*	
H4B	0.797261	0.599555	−0.013743	0.066*	
H4C	0.812511	0.633876	0.095684	0.066*	
C5	0.8601 (7)	0.2841 (7)	0.1252 (4)	0.0333 (11)	
H5A	0.935178	0.317406	0.156388	0.050*	
H5B	0.924538	0.250564	0.055791	0.050*	
H5C	0.839851	0.183013	0.167560	0.050*	
C6	0.4774 (8)	1.0822 (8)	0.1761 (5)	0.0404 (13)	
H6A	0.602434	1.005903	0.189192	0.061*	
H6B	0.461592	1.206568	0.162583	0.061*	
H6C	0.458693	1.046979	0.116050	0.061*	
C7	0.3772 (8)	1.1383 (7)	0.3759 (5)	0.0384 (12)	
H7A	0.299763	1.132681	0.443629	0.058*	
H7B	0.360358	1.261909	0.353195	0.058*	
H7C	0.507049	1.064490	0.380701	0.058*	
C8	0.2159 (9)	0.7503 (8)	0.5644 (5)	0.0450 (15)	
H8A	0.344626	0.694301	0.574038	0.068*	
H8B	0.133983	0.776499	0.631347	0.068*	
H8C	0.194380	0.861157	0.518762	0.068*	
C9	0.2439 (8)	0.4168 (7)	0.6010 (4)	0.0360 (11)	
H9A	0.234863	0.315606	0.580728	0.054*	
H9B	0.165563	0.446406	0.668472	0.054*	
H9C	0.372287	0.385368	0.605036	0.054*	

### Atomic displacement parameters (Å^2^)

**Table:**

	*U*^11^	*U*^22^	*U*^33^	*U*^12^	*U*^13^	*U*^23^
Mo1	0.01869 (18)	0.01643 (18)	0.02248 (19)	−0.00617 (13)	−0.00398 (13)	−0.00316 (13)
S1	0.0218 (5)	0.0294 (6)	0.0292 (6)	−0.0054 (4)	−0.0042 (4)	−0.0106 (5)
S2	0.0237 (5)	0.0172 (5)	0.0394 (7)	−0.0073 (4)	−0.0082 (5)	−0.0026 (5)
S3	0.0216 (5)	0.0270 (6)	0.0252 (6)	−0.0072 (4)	−0.0028 (4)	−0.0029 (5)
O1	0.0227 (17)	0.0234 (17)	0.056 (2)	−0.0051 (13)	−0.0022 (16)	−0.0054 (17)
O2	0.044 (2)	0.047 (2)	0.030 (2)	−0.0096 (18)	−0.0119 (17)	0.0041 (18)
O3	0.040 (2)	0.043 (2)	0.055 (3)	−0.0181 (18)	−0.0106 (19)	−0.023 (2)
O4	0.0189 (14)	0.0297 (17)	0.0239 (16)	−0.0050 (12)	−0.0023 (12)	−0.0042 (13)
O5	0.0325 (17)	0.0176 (15)	0.0384 (19)	−0.0131 (13)	−0.0134 (14)	0.0002 (14)
O6	0.0269 (16)	0.0184 (15)	0.0235 (16)	−0.0079 (12)	−0.0030 (12)	−0.0001 (12)
C1	0.029 (2)	0.0158 (19)	0.030 (2)	−0.0111 (17)	−0.0045 (18)	−0.0030 (17)
C2	0.023 (2)	0.027 (2)	0.029 (2)	−0.0056 (18)	−0.0068 (18)	−0.0029 (19)
C3	0.024 (2)	0.023 (2)	0.034 (3)	−0.0080 (17)	−0.0023 (18)	−0.0117 (19)
C4	0.041 (3)	0.033 (3)	0.041 (3)	−0.008 (2)	0.000 (2)	0.007 (2)
C5	0.024 (2)	0.026 (2)	0.045 (3)	−0.0066 (19)	0.000 (2)	−0.011 (2)
C6	0.047 (3)	0.031 (3)	0.043 (3)	−0.021 (2)	−0.004 (3)	0.002 (2)
C7	0.041 (3)	0.031 (3)	0.049 (3)	−0.017 (2)	−0.005 (2)	−0.014 (2)
C8	0.069 (4)	0.034 (3)	0.035 (3)	−0.027 (3)	0.004 (3)	−0.012 (2)
C9	0.048 (3)	0.030 (3)	0.031 (3)	−0.019 (2)	−0.007 (2)	0.002 (2)

### Geometric parameters (Å, º)

**Table:**

Mo1—C3	1.901 (5)	C4—H4A	0.9800
Mo1—C1	1.915 (5)	C4—H4B	0.9800
Mo1—C2	1.919 (5)	C4—H4C	0.9800
Mo1—O6	2.249 (3)	C5—H5A	0.9800
Mo1—O4	2.264 (3)	C5—H5B	0.9800
Mo1—O5	2.269 (3)	C5—H5C	0.9800
S1—O4	1.518 (3)	C6—H6A	0.9800
S1—C5	1.772 (5)	C6—H6B	0.9800
S1—C4	1.777 (6)	C6—H6C	0.9800
S2—O5	1.516 (3)	C7—H7A	0.9800
S2—C7	1.773 (6)	C7—H7B	0.9800
S2—C6	1.782 (6)	C7—H7C	0.9800
S3—O6	1.522 (3)	C8—H8A	0.9800
S3—C9	1.771 (6)	C8—H8B	0.9800
S3—C8	1.782 (6)	C8—H8C	0.9800
O1—C1	1.174 (6)	C9—H9A	0.9800
O2—C2	1.177 (6)	C9—H9B	0.9800
O3—C3	1.170 (6)	C9—H9C	0.9800
			
C3—Mo1—C1	82.54 (19)	S1—C4—H4C	109.5
C3—Mo1—C2	85.0 (2)	H4A—C4—H4C	109.5
C1—Mo1—C2	85.0 (2)	H4B—C4—H4C	109.5
C3—Mo1—O6	99.49 (18)	S1—C5—H5A	109.5
C1—Mo1—O6	99.79 (17)	S1—C5—H5B	109.5
C2—Mo1—O6	173.76 (16)	H5A—C5—H5B	109.5
C3—Mo1—O4	97.31 (16)	S1—C5—H5C	109.5
C1—Mo1—O4	175.59 (16)	H5A—C5—H5C	109.5
C2—Mo1—O4	99.35 (16)	H5B—C5—H5C	109.5
O6—Mo1—O4	75.88 (12)	S2—C6—H6A	109.5
C3—Mo1—O5	178.08 (18)	S2—C6—H6B	109.5
C1—Mo1—O5	97.90 (16)	H6A—C6—H6B	109.5
C2—Mo1—O5	96.89 (18)	S2—C6—H6C	109.5
O6—Mo1—O5	78.60 (12)	H6A—C6—H6C	109.5
O4—Mo1—O5	82.10 (13)	H6B—C6—H6C	109.5
O4—S1—C5	104.3 (2)	S2—C7—H7A	109.5
O4—S1—C4	104.6 (3)	S2—C7—H7B	109.5
C5—S1—C4	97.9 (3)	H7A—C7—H7B	109.5
O5—S2—C7	104.0 (2)	S2—C7—H7C	109.5
O5—S2—C6	105.7 (2)	H7A—C7—H7C	109.5
C7—S2—C6	98.1 (3)	H7B—C7—H7C	109.5
O6—S3—C9	104.2 (2)	S3—C8—H8A	109.5
O6—S3—C8	106.0 (2)	S3—C8—H8B	109.5
C9—S3—C8	97.2 (3)	H8A—C8—H8B	109.5
S1—O4—Mo1	115.64 (18)	S3—C8—H8C	109.5
S2—O5—Mo1	118.64 (19)	H8A—C8—H8C	109.5
S3—O6—Mo1	120.18 (18)	H8B—C8—H8C	109.5
O1—C1—Mo1	175.0 (4)	S3—C9—H9A	109.5
O2—C2—Mo1	178.2 (5)	S3—C9—H9B	109.5
O3—C3—Mo1	175.6 (4)	H9A—C9—H9B	109.5
S1—C4—H4A	109.5	S3—C9—H9C	109.5
S1—C4—H4B	109.5	H9A—C9—H9C	109.5
H4A—C4—H4B	109.5	H9B—C9—H9C	109.5
			
C5—S1—O4—Mo1	−167.4 (2)	C6—S2—O5—Mo1	−94.2 (3)
C4—S1—O4—Mo1	90.3 (3)	C9—S3—O6—Mo1	164.8 (2)
C7—S2—O5—Mo1	163.1 (2)	C8—S3—O6—Mo1	−93.3 (3)

### Hydrogen-bond geometry (Å, º)

**Table:**

*D*—H···*A*	*D*—H	H···*A*	*D*···*A*	*D*—H···*A*
C5—H5*C*···O1^i^	0.98	2.52	3.461 (7)	161
C4—H4*C*···O3^ii^	0.98	2.60	3.503 (8)	154
C7—H7*C*···O1^ii^	0.98	2.51	3.419 (7)	154
C9—H9*C*···O5^iii^	0.98	2.45	3.239 (7)	138
C4—H4*B*···O3^iv^	0.98	2.38	3.334 (8)	165
C9—H9*A*···O1^v^	0.98	2.59	3.328 (7)	132
C9—H9*B*···O3^v^	0.98	2.52	3.469 (7)	163
C7—H7*B*···O6^vi^	0.98	2.55	3.398 (6)	144

Symmetry codes: (i) *x*+1, *y*−1, *z*; (ii) *x*+1, *y*, *z*; (iii) −*x*+1, −*y*+1, −*z*+1; (iv) −*x*+1, −*y*+1, −*z*; (v) −*x*, −*y*+1, −*z*+1; (vi) *x*, *y*+1, *z*.
